# Vaccine coverage and compliance in Mexico with the two-dose and three-dose rotavirus vaccines

**DOI:** 10.1080/21645515.2018.1540827

**Published:** 2018-12-11

**Authors:** Gerardo Luna-Casas, Roberto Carreño-Manjarrez, Andrés Castañeda-Prado, Maria Yolanda Cervantes-Apolinar, Ricardo Navarro-Rodriguez, Gilberto Sánchez-González, Ricardo Cortés-Alcalá, Rodrigo DeAntonio

**Affiliations:** a Estimatio SC, Mexico, Mexico; b GSK, City of Knowledge, Clayton, Ancon, Panama; c GSK, Calz México-Xochimilco, Mexico City, Mexico; d GSK, Mexico City, Mexico

**Keywords:** *Rotarix*, *RotaTeq*, rotavirus, Mexico, dosing, coverage, compliance

## Abstract

Worldwide, rotavirus infection has been a leading cause of severe diarrhea morbidity and mortality. Two rotavirus vaccines have been used in the National Immunization Program (NIP) in Mexico; two-dose *Rotarix* from 2006 to 2011 and three-dose *RotaTeq* since 2011. This study assessed coverage (receiving at least one dose or full dose series) in eligible infants, compliance (% completing dose series and % completing series on schedule) in eligible infants vaccinated with *Rotarix* (2010) versus *RotaTeq* (2012), using Mexican Social Security Institute data nationwide and by regions.

In 2010, 80.7% received at least one dose of *Rotarix*, 75.6% received both doses and 57.0% received both doses on schedule. In 2012, 85.7% received at least one dose of *RotaTeq*, 61.0% received all three doses and 43.2% received all three doses on schedule. More eligible infants received all doses with *Rotarix* versus *RotaTeq* (p < 0.001). Among infants vaccinated with *Rotarix* versus *RotaTeq*, 93.7% versus 71.1% completed full series (p < 0.001), and 75.5% versus 70.9% completed full series on schedule (p = 0.105), respectively. The full series coverage and compliance decreased in all regions with *RotaTeq* compared with *Rotarix*. In conclusion, rotavirus vaccination has successfully reduced morbidity and mortality in children under 5 years in Mexico. This study found significant differences in full series coverage and compliance among infants and a higher proportion of completed scheduled at an earlier age in Mexico when comparing a two-dose vaccine in 2010 with a three-dose vaccine in 2012. Such differences might need to be taken into consideration to maximize NIP benefits, including early protection of the rotavirus vaccination program.

## Introduction

Worldwide, rotavirus infection affects almost all children by the age of five. Though many cases have mild features with prompt recovery, some cases can lead to severe forms including dehydration which is the most common cause of associated death.^1^ Rotavirus is the leading cause of severe diarrhea, causing around 215,000 deaths in young children globally in 2013.^2^ By 2014, more than 70 countries included rotavirus vaccines in their national immunization program (NIP), increasing to 95 countries offering some form of rotavirus vaccine program by 2018.^3^ As a result, significant reductions in hospitalizations and deaths are observed (e.g., Mexico, Brazil and Panama reported 22–50% fewer diarrhea-related deaths in young children).^2^

In Mexico, prior to rotavirus vaccination, diarrheal disease was responsible for around 5% of deaths in children under five years, and 3,000 deaths per year.^4^ Introducing two-dose rotavirus vaccine (*Rotarix*, GSK) in the NIP of Mexico in 2007 substantially reduced mortality and morbidity in children under five, regardless of differences by region or socioeconomic status. Mortality related to diarrhea declined by 35% the three years after introducing rotavirus in the NIP as well as an 40% reduction in hospitalizations.^5–7^

Sustained yearly decreases in mortality of 53% and morbidity of 47% were also reported after seven year post vaccine introduction in the NIP, resulting in 959 deaths and 5,831 hospitalizations averted annually post vaccine introduction among children under 5 years reported by Sánchez-Uribe *et al*.^8^

In addition to two-dose *Rotarix* (administered at 2 and 4 months of age), three-dose *RotaTeq* (Merck) is available, administered at 2, 4, and 6 months of age.^9–11^ *Rotarix* was available on the private market in Mexico since 2005, implemented in the NIP in some areas in 2006 and nationally in 2007.^12^ Since 2011, the NIP uses *RotaTeq*.^13^ Both *Rotarix* and *RotaTeq* have good efficacy and safety profiles, supported by extensive data.^1,14–16^

Due to differences in the number of doses between the vaccines, this study assessed coverage and compliance achieved with *Rotarix* (in 2010) and *RotaTeq* (in 2012), using national public data from the Mexican Social Security Institute (Instituto Mexicano del Seguro Social, Spanish acronym: IMSS) database of eligible and vaccinated Mexican infants. Outcomes for coverage included the proportion of eligible infants who received at least one dose (i.e., one-dose coverage) and the full series (i.e., full-series coverage). Outcomes for compliance included the proportion of vaccinated infants who received the full series (i.e., series completion) and who received the full series at the age recommended by the NIP and dose intervals (i.e., series timeliness). Data by gender, vaccine schedule, age, state and region were also compared.

## Results

**Figure 1.**
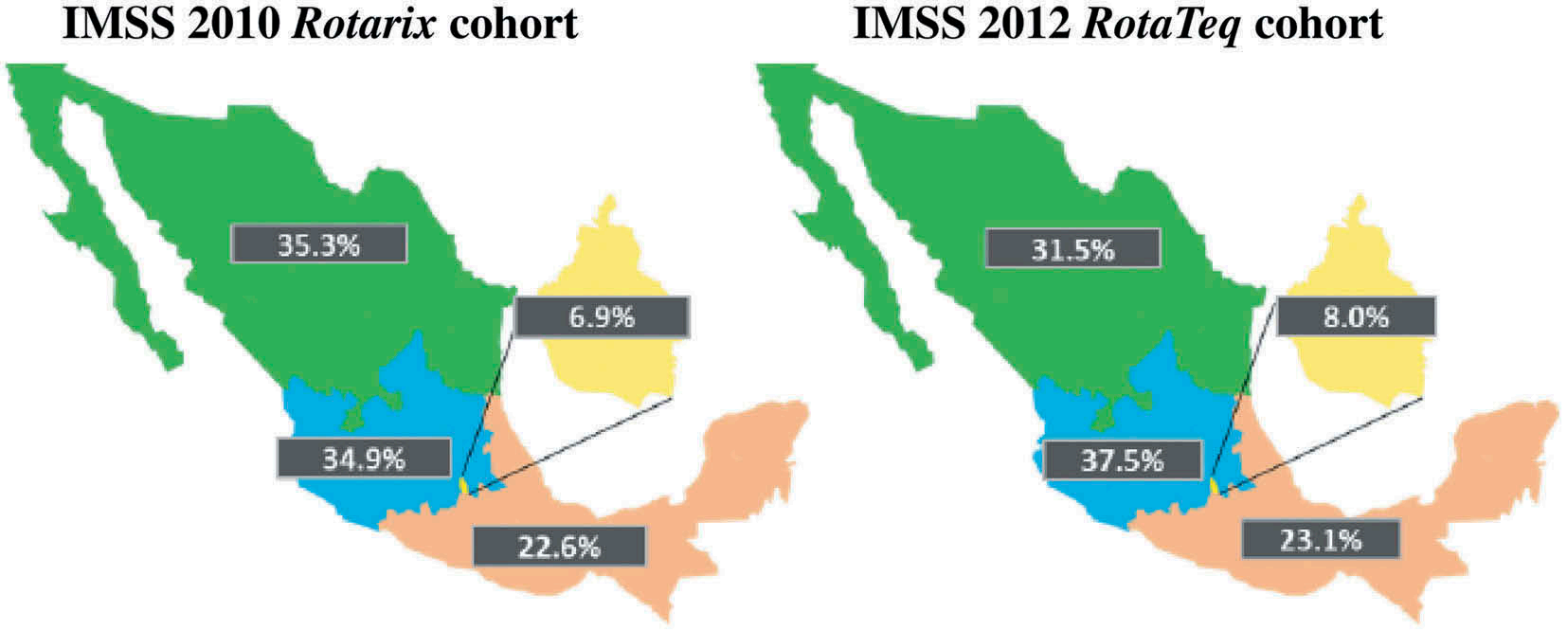
Distribution of *Rotarix* and *RotaTeq* eligible infants (%) by region. IMSS: Instituto Mexicano del Seguro Social; *Rotarix*: two-dose rotavirus vaccine; *RotaTeq*: three-dose rotavirus vaccine; Green: North region; Blue: Central region; Pink: South region; Yellow: Mexico City.

The IMSS registry included 659,249 infants (50.7% male) eligible for *Rotarix* in 2010 and 780,483 infants (51.2% male) eligible for *RotaTeq* in 2012. Most eligible infants were located in the North and Central regions (Figure 1). The registry considered the working force population and their families associated with the IMSS in 2010, and in 2012 also included the population from the *Oportunidades* Program, in addition to the standard IMSS population.

### Vaccine coverage

**Figure 2.**
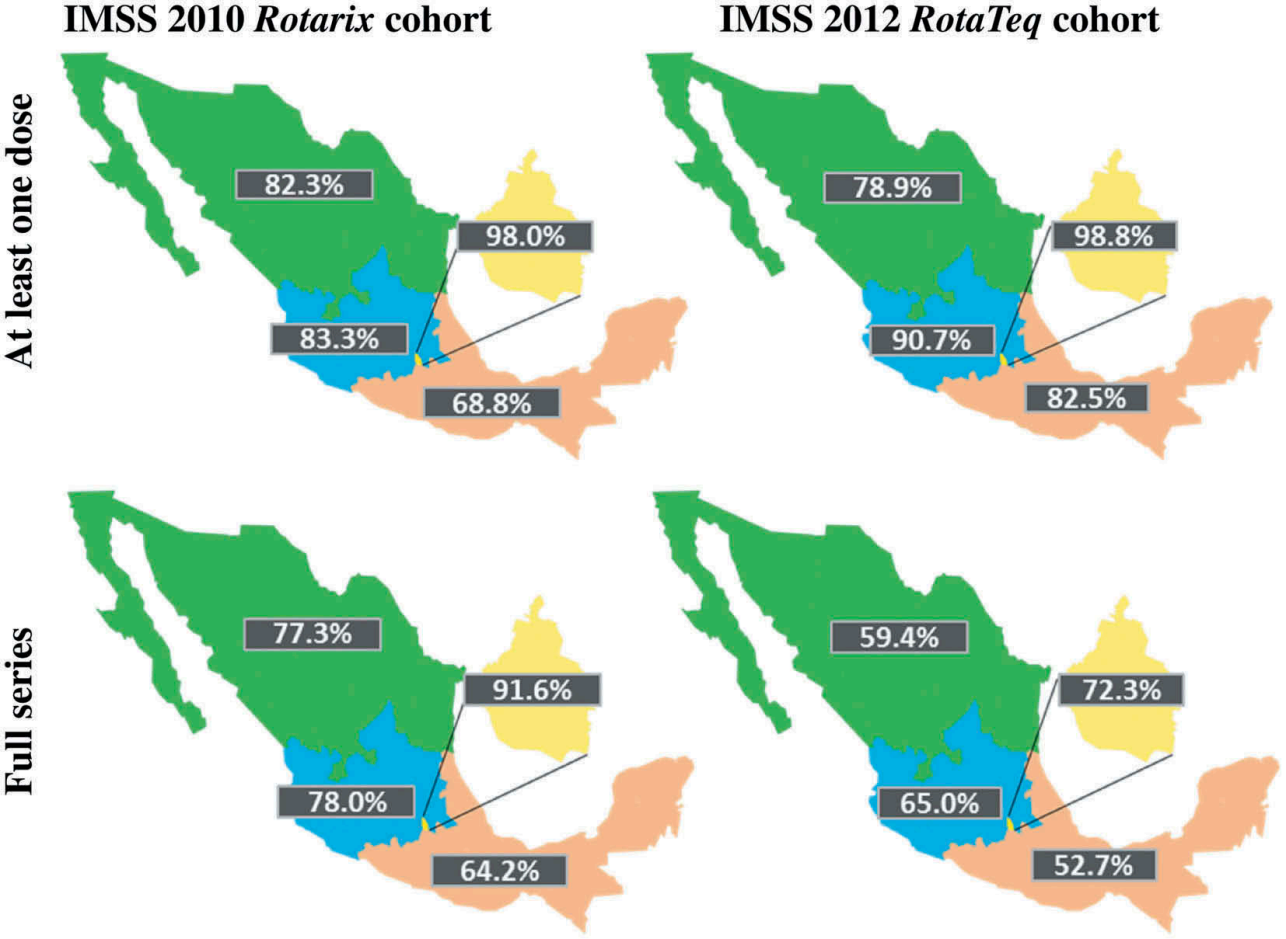
*Rotarix* and *RotaTeq* vaccine coverage (at least one dose and full series) by region. IMSS: Instituto Mexicano del Seguro Social; *Rotarix*: two-dose rotavirus vaccine; *RotaTeq*: three-dose rotavirus vaccine; Green: North region; Blue: Central region; Pink: South region; Yellow: Mexico City.

The 2010 cohort vaccinated with at least one dose of *Rotarix* comprised 532,082 infants (51.3% male) and the 2012 cohort vaccinated with at least one dose of *RotaTeq* comprised 668,958 infants (51.0% male). The national coverage of infants receiving at least one dose was 80.7% with *Rotarix* and 85.7% with *RotaTeq* (p = 0.11), and was similar between male and female infants, respectively (i.e., 81.7% and 79.7% for *Rotarix*, and, 85.4% and 86.1% for *RotaTeq*) (Table 1). Regional coverage with *Rotarix* ranged from 68.8% in the South to 98.0% in Mexico City, and with *RotaTeq* from 78.9% in the North to 98.8% in Mexico City (Figure 2).

**Table 1.** *Rotarix* and *RotaTeq* vaccine coverage (infants receiving at least one dose or full series out of all eligible infants) by sex and region.

Vaccine coverage among vaccine-eligible children							
	*Rotarix* (N = 659,249)		*RotaTeq* (N = 780,483)		*Rotarix* vs. *RotaTeq*		
	n	%	n	%	n	%	p
**At least one dose coverage**	**532,082**	**80.7**	**668,958**	**85.7**	**136,876**	**5**	**0.11**
**By sex:**							
Male	272,981	81.7	341,220	85.4	68,239	3.6	0.21
Female	259,101	79.7	327,738	86.1	68,637	6.4	0.04
**By region:**							
North	193,285	82.3	193,577	78.9	292	−3.5	0.39
Center	191,668	83.3	265,165	90.7	73,497	7.3	0.02
Mexico City	44,804	98.0	61,497	98.8	16,693	0.8	0.11
South	102,325	68.8	148,719	82.5	46,394	13.7	0.01
**Full series coverage**	**498,312**	**75.6**	**475,845**	**61.0**	**−22,467**	**−14.6**	**<0.001**
**By sex:**							
Male	256,513	76.8	242,693	60.7	−13,820	−16.1	<0.001
Female	241,799	74.4	233,152	61.2	−8,647	−13.1	<0.001
**By region:**							
North	181,382	77.3	145,714	59.4	−35,668	−17.9	<0.001
Center	179,471	78.0	190,045	65.0	10,574	−13.1	<0.001
Mexico City	41,902	91.6	45,018	72.3	3,116	−19.3	<0.001
South	95,557	64.2	95,068	52.7	−489	−11.5	<0.001

*Rotarix*: two-dose rotavirus vaccine; *RotaTeq*: three-dose rotavirus vaccine; N: total number of vaccinated infants; n: number in subcategory; Chi square proportion comparison; p value. Full series coverage: Number of vaccine-eligible infants that received full series of rotavirus vaccines regardless of timeliness. At least one dose coverage: Number of vaccine-eligible infants that received at least one dose of rotavirus vaccine regardless of timeliness.

The number of infants who received the full vaccination series was 498,312 for *Rotarix* and 475,845 for *RotaTeq*, which corresponds to an estimated full series coverage of 75.6% with *Rotarix* and 61.0% with *RotaTeq* (p < 0.001). The full series coverage was comparable for male and female infants (Table 1). Regional differences existed, with full series coverage ranging from 64.2% to 91.6% with *Rotarix*, and 52.7% to 72.3% with *RotaTeq* in the South versus Mexico City, respectively (Figure 2).

### Vaccine compliance

**Figure 3.**
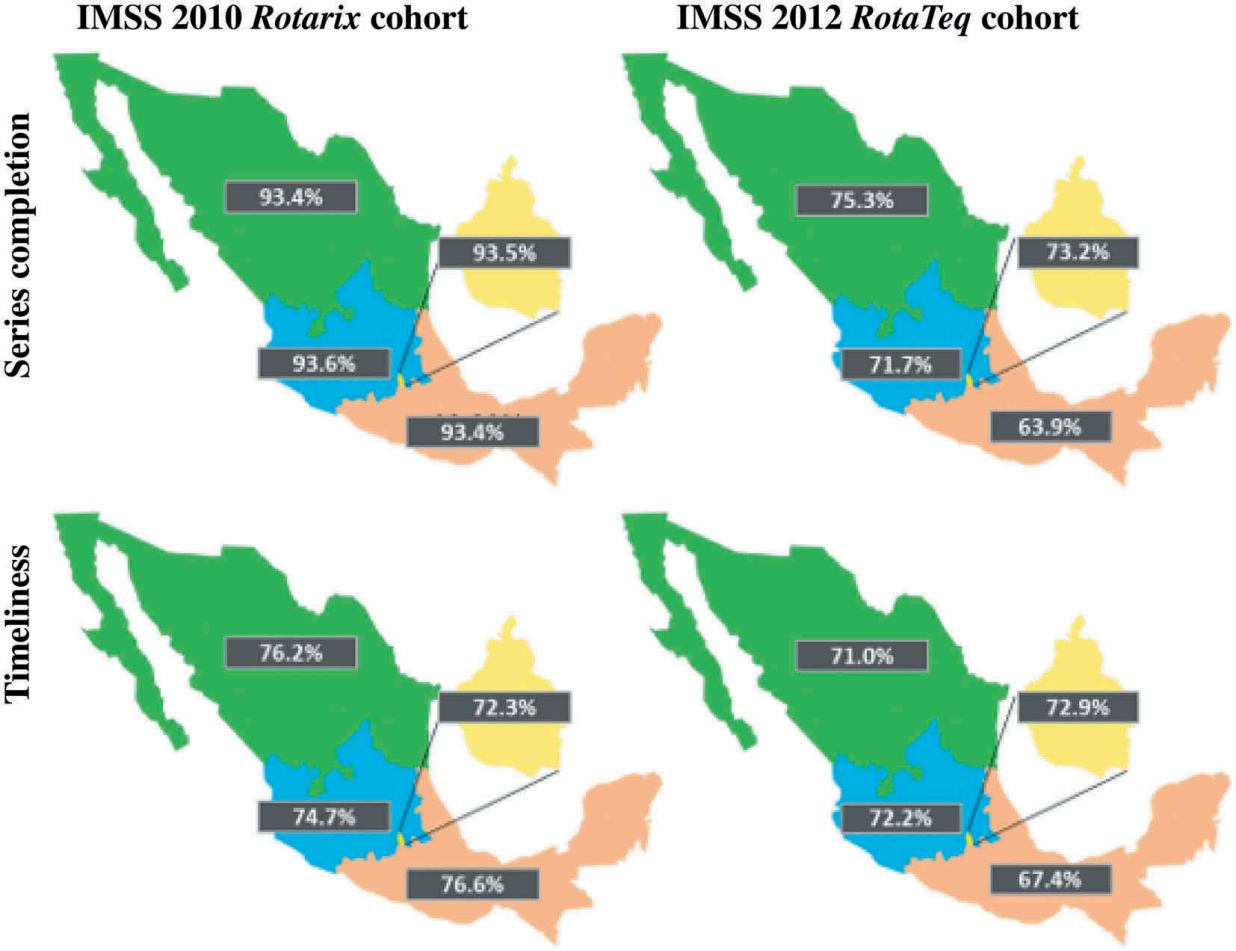
*Rotarix* and *RotaTeq* compliance (series completion, timeliness) by region. IMSS: Instituto Mexicano del Seguro Social; *Rotarix*: two-dose rotavirus vaccine; *RotaTeq*: three-dose rotavirus vaccine; Green: North region; Blue: Central region; Pink: South region; Yellow: Mexico City.

The number of infants who completed the full series among those vaccinated was 498,312 (93.7%) with *Rotarix* and 475,845 (71.1%) with *RotaTeq* nationally (p < 0.001). Compliance was consistently high throughout the country with *Rotarix* but varied considerably across regions with *RotaTeq* (Figure 3, Table 2): the highest compliance was observed in the North (75.3%) and the lowest in the South (63.9%).

**Table 2.** *Rotarix* and *RotaTeq* compliance (infants with series completion and timeliness) among *Rotarix*- or *RotaTeq*-vaccinated infants and among all eligible infants.

Vaccine compliance among vaccinated infants							
	*Rotarix* (N = 532,082)		*RotaTeq* (N = 668,958)		*Rotarix* versus *RotaTeq*		
	n	%	n	%	n	%	p
**Full series completion**	**498,312**	**93.7**	**475,845**	**71.1**	**−22,467**	**−22.5**	**<0.001**
**By sex:**							
Male	256,513	94.0	242,693	71.1	−13,820	−22.8	<0.001
Female	241,799	93.3	233,152	71.1	−8,647	−22.2	<0.001
**By region**							
North	181,382	93.4	145,714	75.3	−35,668	−18.6	<0.001
Center	179,471	93.6	190,045	71.7	10,574	−22.0	<0.001
Mexico City	41,902	93.5	45,018	73.2	3,116	−20.3	<0.001
South	95,557	93.4	95,068	63.9	−489	−29.5	<0.001
**Full series timeliness**	**375,992**	**75.5**	**337,424**	**70.9**	**−38,568**	**−4.5**	**0.105**
**By sex:**							
Male	195,405	76.2	174,695	72.0	−20,710	−4.2	0.36
Female	180,587	74.7	162,729	69.8	−17,858	−4.9	0.31
**By region:**							
North	136,313	75.2	103,395	71.0	−32,918	−4.2	0.52
Center	136,231	75.9	137,126	72.2	895	−3.8	0.48
Mexico City	30,290	72.3	32,810	72.9	2,520	0.6	0.95
South	73,158	76.6	64,093	67.4	−9,065	−9.1	0.23
Vaccine compliance among vaccine-eligible infants							
	*Rotarix* (N = 659,249)		*RotaTeq* (N = 780,483)		*Rotarix* versus *RotaTeq*		
	n	%	n	%	n	%	p
Full series coverage	498,312	75.6	475,845	61.0	−22,467	−14.6	**<0.01**
Full series coverage timeliness	375,992	57.0%	337,424	43.2%	−38,568	−13.8	**<0.01**
Vaccine timeliness characteristics among infants with full series coverage timeliness							
	*Rotarix*		*RotaTeq*		*Rotarix* versus *RotaTeq*		
	(N = 375,992)		(N = 337,424)				n
Age at last dose (Mean weeks)	14.6		19.8		5.2		
Interval 1st to last dose (Mean weeks)	5.1		10.3		5.2		

*Rotarix*: two-dose rotavirus vaccine; *RotaTeq*: three-dose rotavirus vaccine; n: number in subcategory; Chi square proportion comparison; p value. Full series completion: Number of vaccination infants who completed all recommended doses regardless of timeliness. Full series timeliness: number of vaccinated infants who completed all doses as per the NIP guidance at recommended age and intervals, Full series coverage: Number of vaccine-eligible infants that received full series of rotavirus vaccines regardless of timeliness. At least one dose coverage: Number of vaccine-eligible infants that received at least one dose of rotavirus vaccine regardless of timeliness; Full series coverage timeliness: number of vaccine-eligible infants who completed all doses as per the NIP guidance at recommended age and intervals.

The mean age to complete the *Rotarix* last dose was 14.6 months while the mean age for completing *RotaTeq* was 19.8 months (p < 0.01) (see Supplementary file 1).

The number of infants who completed the full series at the designated age and correct dosing intervals among those vaccinated (i.e., compliance timeliness among vaccinated infants) was 375,992 (75.5%) with *Rotarix* and 337,424 (70.9%) with *RotaTeq* (p = 0.105). There was more consistency across regional rates among those that complied with the full dosing schedule on time, with a range of 72.3–76.6% with *Rotarix* and 67.4–72.9% with *RotaTeq* (Figure 3). When comparing compliance timeliness among the full eligible population (both vaccinated and unvaccinated infants), the difference in compliance between *Rotarix* and *RotaTeq* is even more pronounced: 57.0% compliance with *Rotarix* versus 43.2% with *RotaTeq*, a resulting decrease of nearly 14% (p < 0.01) (Table 2).

### Early protection

Among infants who initiated rotavirus vaccination, 29.3% of infants by 16 weeks of age had already completed the full series with *Rotarix* compared to 14.3% with *Rotateq* (p < 0.01). By 16 weeks of age,29.3% of infants had completed the full series with *Rotarix* while14.3% with *RotaTeq* (p < 0.01). Additionally, 31.2% and 26.1% completed the full series by24 weeks of age for *Rotarix* and *RotaTeq*, respectively (p < 0.05) (Table 3); similar trends were observed in the vaccine eligible infant population

**Table 3.** Rotarix and RotaTeq early protection in vaccine eligible and vaccinated infants.

Early protection among vaccine eligible infants						
	*Rotarix*		*RotaTeq*			
	*(N = 659,249)*		(N = 780,483)		*Rotarix* vs. *RotaTeq* difference	
	n	%	n	%	%	p
**Full series coverage by age:**						
16 WoA	155,806	23.6%	95,340	12.2%	−11.4%	< 0.01
24 WoA	165,994	25.2%	174,452	22.4%	−2.8%	< 0.05
32 WoA	176,512	26.8%	206,052	26.4%	−0.4%	> 0.05
Early protection among vaccinated infants						
	*Rotarix*		*RotaTeq*			
	*(N = 532,082)*		(N = 668,958)		*Rotarix* vs. *RotaTeq*	
	n	%	n	%	%	p
**Full series completion by age:**						
16 WoA	155,806	29.3%	95,340	14.3%	−15.0%	<0.01
24 WoA	165,994	31.2%	174,452	26.1%	−5.1%	<0.05
32 WoA	176,512	33.2%	206,052	30.8%	−2.4%	<0.05

WoA: weeks of age (age in weeks)

Full series coverage by age: the number of infants who received all doses by that age divided by the number of vaccine eligible infants

Full series completion by age: the number of infants who received all doses by that age divided by the number of vaccinated infants

### Comparison with full series coverage of DTaP+ IPV+ Hib

**Figure 4.**
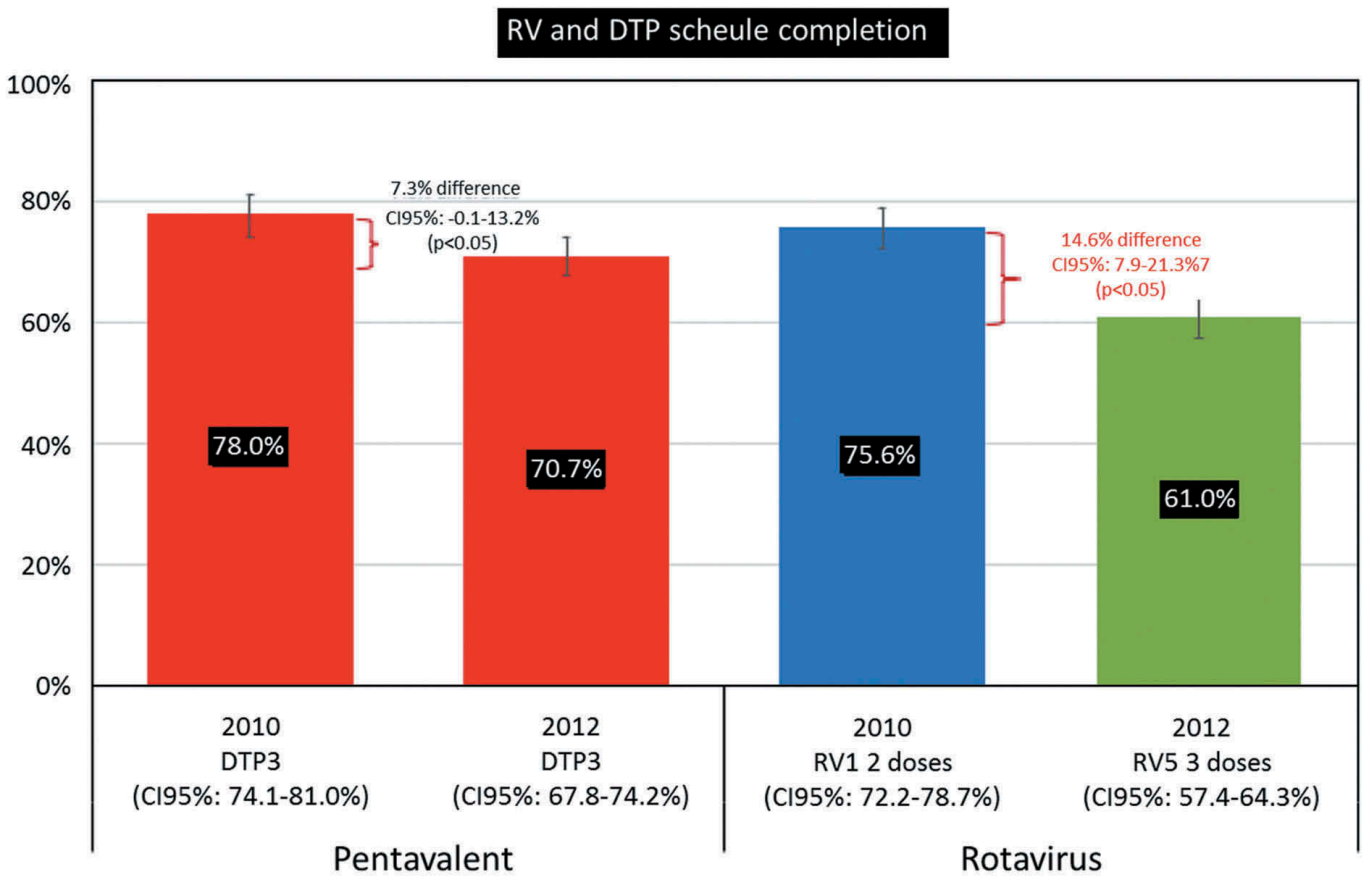
Full series coverage of DTaP+ IPV+ Hib and rotavirus vaccines in 2010 and 2012. DTaP3: DTaP+ IPV+ Hib three-dose vaccine; *Rotarix*: two-dose rotavirus vaccine; *RotaTeq*: three-dose rotavirus vaccine. Full series coverage: Number of vaccine-eligible infants that received full series of rotavirus vaccines regardless of timeliness.

The full series coverage of pentavalent DTaP+ IPV+ Hib vaccine was 78.0% in 2010 and 70.7% in 2012, giving a difference of −7.3% in 2012 versus 2010, however, this difference was not statistically significant (p = 0.06) (Table 4, Figure 4). The largest changes in coverage were observed in the North and in Mexico City with a difference in full series coverage in 2012 versus 2010 of −12.2% and −13.2%, respectively). Full series coverage also decreased within the rotavirus vaccination program between 2010 and 2012 but to a much larger degree than observed with the DTaP+ IPV+ Hib vaccine and was statistically significant (difference of −14.6%, p < 0.001) (Table 4).

**Table 4.** Comparison of full series coverage with DTaP+ IPV+ Hib and rotavirus program between 2010 to and 2012.

Vaccine coverage among vaccine-eligible infants							
	2010 (N = 659,249)		2012 (N = 780,483)		2010 vs. 2012		
	n	%	n	%	n	%	*p*
**Full series coverage DTaP+ IPV+ Hib**	**514,328**	**78.0**	**551,978**	**70.7**	**37,650**	**−7.3**	**0.06**
**By region:**							
North	189,204	80.6	167,911	68.4	−21,293	−12.2	<0.001
Center	183,556	79.8	213,973	73.2	30,417	−6.7	0.12
Mexico City	42,867	93.8	50,166	80.6	7,299	−13.2	0.06
South	98,701	66.3	119,928	66.5	21,227	0.2	0.97
**Full series coverage Rotavirus program**	**498,312**	**75.6**	**475,845**	**61.0**	**−22,467**	**−14.6**	**<0.001**
**By region:**							
North	181,382	77.3	145,714	59.4	−35,668	−17.9	<0.001
Center	179,471	78.0	190,045	65.0	10,574	−13.1	<0.001
Mexico City	41,902	91.6	45,018	72.3	3,116	−19.3	<0.001
South	95,557	64.2	95,068	52.7	−489	−11.5	<0.001

IMSS: Mexican Social Security Institute (Instituto Mexicano del Seguro Social); DTaP+ IPV+ Hib: pentavalent DTaP+ IPV+ Hib vaccine against diphtheria, tetanus, pertussis, polio and *Haemophilus* influenzae type b; *Rotarix*: two-dose rotavirus vaccine; *RotaTeq*: three-dose rotavirus vaccine; n: number in subcategory; Chi square proportion comparison; *p value*. Full series coverage: Number of vaccine-eligible infants that received full series of rotavirus vaccines regardless of timeliness.

## Discussion

Due to differences in the scheduled number of doses between *Rotarix* and *RotaTeq*, the vaccine coverage and compliance achieved with each vaccine were compared. Vaccine coverage for infants receiving at least one dose of vaccine was higher in 2012 with *RotaTeq* than in 2010 with *Rotarix* (85.7% versus 80.7%) nationally, mainly due to increases in coverage in the Center region (83.3% to 90.7%) and the South region (68.8% to 82.5%). The improvement in first-dose coverage may reflect an increased acceptance of the rotavirus vaccination program in 2012. Vaccine coverage for infants completing the full series, however, was higher with *Rotarix* than *RotaTeq* (75.6% versus 61.0%) nationally and in all regions; i.e., full series coverage decreased between 2010 and 2012 in the North (77.3% to 59.4%), Mexico City (91.6% to 72.3%), the Center (78.0% to 65.0%) and the South (64.2% to 52.7%). Despite efforts to assure vaccine coverage by the NIP, significantly more *Rotarix*-vaccinated infants compared with *RotaTeq*-vaccinated infants completed the full series (93.7% versus 71.1%) and completed the full series according to the recommended age and dose intervals (57.0% versus 43.2% among all eligible infants). Such differences were even more pronounced within the Southern region, aless developed areas, which had the lowest full series coverage (with *Rotarix* and *RotaTeq*) and compliance (with *RotaTeq*), highlighting the need to strengthen existing vaccination programs and implement strategies to increase vaccination particularly in these vulnerable areas. Improving coverage and timely delivery of vaccinations will result in more health benefits from the vaccination program for both vaccinated individuals and the population at large. When vaccine coverage is low or when timely compliance with the full series is not achieved, infants are left vulnerable to infectious diseases and their negative impact on their health.^17^

The findings of this study are in line with other studies from the US which reported suboptimal coverage of rotavirus vaccination despite the availability of two effective vaccines. Coverage and compliance with two-dose *Rotarix* were, however, higher than with three-dose *RotaTeq*. One US study found that 91% of those receiving *Rotarix* received all doses compared with 83% receiving *RotaTeq* (p < 0.001), and that 75% on *Rotarix* were fully compliant with the recommended dosing schedule versus 60% on *RotaTeq* (p < 0.001).^18^ Better series completion rates and schedule compliance rates were also found for *Rotarix* versus *RotaTeq* in studies of commercially-insured US populations; e.g., 85% of *Rotarix* versus 76% of *RotaTeq* vaccinated infants completed the full series at any time, and 69% *Rotarix* versus 54% *RotaTeq* completed on schedule (both comparisons statistically significant).^19^ Similarly, another study found significantly more commercially-insured US infants receiving *Rotarix* completed the full series than *RotaTeq* or a mix of both vaccines (87% versus 79% versus 73%).^20^ Among Medicaid-covered infants, significantly higher completion (65% versus 46%) and compliance (65% versus 31%) were observed with *Rotarix* than *RotaTeq*, respectively.^21^ The results also highlight the advantage of *Rotarix* two dose schedule in offering early protection by assuring most children will have the recommended schedule before 24 weeks of age, which has been associated with improving the vaccine effectiveness for rotavirus vaccination as recommended by the WHO.^22^

To explore possible temporal biases on vaccination coverage between *Rotarix* cohort in 2010 and the *Rotateq* cohort in 2012, we evaluated the pentavalent DTaP+ IPV+ Hib vaccine coverage. As this vaccine is one of the most homogenous and stable vaccination programs in Latin America it was considered a good reference for the assessment of change in general coverage and rates over time. In 2010, the full series vaccine coverage for the pentavalent vaccine was 78.0% decreasing to 70.7% in 2012 (p = 0.06) nationally but not statistically significant. which led to the conclusion that other driving factors besides general coverage decreases in the NIP (i.e., differences between *Rotarix* and *RotaTeq* dosing) also contributed to the decreased coverage of the rotavirus program between 2010 and 2012.

An increase in the eligible population of approximately 120,000 was observed in 2012 compared to 2010 due to the expansion of the IMSS registry to include coverage of infants from vulnerable population living in rural or marginal urban areas in 2012. Although we observed these differences in the population, when we evaluate the pentavalent DTaP+ IPV+ Hib vaccine coverage in 2010 and 2012, the vaccine coverage was similar suggesting minimal bias on vaccination coverage due to difference in eligible population in 2010 vs 2012.

Rotavirus vaccination has already dramatically reduced the burden of disease in Mexico by reducing childhood deaths and hospitalizations due to diarrhea. The challenge is now to reverse the decreasing coverage and compliance rates observed in all geographic regions, in order to maximize protection against this vaccine-preventable disease. A systematic review of vaccination compliance issues in developed countries identified a range of significant factors in families and in healthcare systems that had a negative effect on compliance. Some key factors influencing poor compliance in families were: low socioeconomic status, no health insurance, low parental education, lack of knowledge about disease and vaccination, younger maternal age, and forgetting vaccination appointments. Some healthcare system factors contributing to poor compliance were: inadequate support, lack of health structures, doubts about medical information, and accessibility issues.^23^ To address some of these barriers to vaccination, educational programs could be implemented to address knowledge gaps for parents as well as healthcare providers, healthcare systems can record and remind patients about vaccinations, and immunization services can be integrated into other healthcare sites to improve access.^24^

There are several limitations to this analysis. This was a retrospective database analysis that relied on IMSS data generated for the institution’s administrative and statistical purposes and not for the specific objectives of this study. Therefore, despite covering a large proportion of the country’s population, the data may be subject to omissions or errors of capture and record bias. The study compared data from one year only (2010 versus 2012) for each vaccine rather than longer term trends. Therefore, it was not possible to assess the impact on coverage increases over time with the two-dose versus the three-dose vaccine. Even though implementation time was shorter for *Rotateq*, the rotavirus vaccination program was already fully established and running in the year of this assessment. Additionally, no supplies issues were reported during the years evaluated.

Because of the upper age restriction for rotavirus vaccination linked to the risk of intussusception, there is only a limited window to vaccinate these infants and therefore, compliance and timeliness is even more important in the case with RV vaccine. As seen in the data, providers appeared to follow the strict recommendations about this window and since this is even smaller for 3rd dose, it results in many of the *Rotateq* children not receiving full schedule and not able to achieve the recommended protection against rotavirus disease.

## Conclusion

Rotavirus vaccination has successfully reduced morbidity and mortality in children under 5 years in Mexico since the introduction of a NIP in 2007.

This study found significant differences in full series coverage and compliance and a higher proportion of completed scheduled at an earlier age among infants in Mexico when comparing a two-dose vaccine in 2010 with a three-dose vaccine in 2012. Such differences might need to be taken into consideration to maximize NIP benefits, including early protection, of the rotavirus vaccination program.

## Patients and methods

Data from all infants in the IMSS registry aged 2 to 8 months and who received their first dose of *Rotarix* vaccine between January 1 and December 31, 2010, or their first dose of *RotaTeq* between January 1 and December 31, 2012 was included. Data was reviewed till February 2011 for *Rotarix* and March 2013 for *Rotateq* for infants whom received the first dose in the second half of the study year to allow enough time for infants to be age eligible to receive all doses of each vaccine. Those with incomplete data were excluded from the analysis.

The data collected were the date of birth, gender, residential state, rotavirus vaccine, total number of doses, and the date of each dose. The following databases were accessed to retrieve these data; Integral Healthcare Information System (Sistema Informático de Atención Integral a la Salud del IMSS, SIAIS), Yearbooks IMSS, National Health Information System (Sistema Nacional de Información en Salud, SINAIS), and National Transparency Platform (Plataforma Nacional de Transparencia, PNT).

The outcomes assessed were: vaccine coverage (defined as the number of infants vaccinated with one dose or the full series divided by the number of eligible infants) and vaccine compliance (defined as the number of infants who received all doses (full series completion) or received all doses at the recommended time (full series timeliness) divided by the number of vaccinated infants) (see Supplementary file 2).

Findings were presented at the national level and stratified by region (i.e., North, Central, South and Mexico City), as previously done in the 2012 National Health and Nutrition Survey (see Supplementary file 3).^25^ The outcomes were also analyzed by gender, rotavirus vaccine, age, and state.

For compliance and timeliness outcomes, the following recommendations were considered:

- *Rotarix* is a two-dose oral vaccine; the first dose can be administered between six and 20 weeks of age (eight weeks is the practice at IMSS), with a minimum interval of four weeks before the second dose, and a schedule completion date before the age of 24 weeks.^10,26^
- *RotaTeq* is a three-dose oral vaccine; the first dose can be administered between six and 12 weeks of age (eight weeks is the practice at IMSS), with the subsequent doses administered at 4-to-10-week intervals. The third dose should not be given after 32 weeks of age.^11,26^

All variables collected were analyzed descriptively using STATA version 14.1; numbers and percentages for dichotomous and polychotomous variables, means, medians and percentiles, results stratified by analytic period and bivariate comparisons of outcome measures by cohort (*Rotarix* vs *RotaTeq*), and by state and geographic region. For each analytic period, the number of eligible recipients was tabulated by age, gender and number of vaccine doses completed. Time and attrition between doses was examined by tabulating the number of infants by the number of *Rotarix* and *RotaTeq* doses completed. Compliance was assessed during each analytic period by comparing the proportion of infants who received their respective vaccination on-schedule, and the proportion completing their respective vaccination series.

Proportions of cases achieving each outcome in the *Rotarix* versus *RotaTeq* groups were compared using the Chi-squared test, as recommended by Campbell (2007)^27^ and Richardson (2011),^28^ and p values were calculated, according to the recommended method given by Altman *et al*. (2000).^29^

### Temporal bias assessment

The study compared 2010 data for *Rotarix* to 2012 data for *RotaTeq*, therefore potential temporal bias needed to be assessed. As such, changes in coverage with the pentavalent DTaP+ IPV+ Hib vaccine between 2010 and 2012 (using the same data sources) were analyzed and compared to changes in coverage with the rotavirus program. If similar coverage changes are observed, then differences between *Rotarix* and *RotaTeq* are likely to be associated with operational factors of the program, rather than differences in the number of doses.

## Supplementary Material

Supplemental Material

## Declarations

### Trademark statement

*Rotarix* is a trademark of the GSK group of companies.

*RotaTeq* is a trademark of Merck & Co., Inc.

## Patient highlights

- Rotavirus infection affects most young children worldwide, causing diarrhea which can sometimes lead to severe dehydration and death. Many countries, including Mexico, now protect infants with either a two-dose (*Rotarix*) or three-dose (*RotaTeq*) vaccination schedule.
- This study looked at how many infants received the complete dose series of *Rotarix* (in 2010) or *RotaTeq* (in 2012), and whether they received them at the recommended age to have optimal protection from the vaccine.
- Among infants who were eligible to be vaccinated, many more received the full series of doses with *Rotarix* than with *RotaTeq*: 75.6% versus 61.0%.
- Among infants who initiated rotavirus vaccination, many more who received *Rotarix* got the full series of doses compared with those who received *RotaTeq* (93.7% versus 71.1%), and 75.5% versus 70.9% completed the full series according to the schedule recommended by the NIP.
- A higher proportion of infants who initiated rotavirus vaccination were able to complete the full schedule at an earlier age with *Rotarix* compared to *Rotateq*: 29.3% versus 14.3% by 16 weeks of age, 31.2% versus 26.1% by 24 weeks of age and 33.2% and 30.8% by 32 weeks of age

Since Mexico switched from using *Rotarix* to *RotaTeq* in 2011, this study found that fewer eligible infants received the full series of doses, and fewer vaccinated infants completed the full series or completed it according to recommendations. This reduction was seen across all geographic regions of the country.
