# Supplementary material for: Vaccine coverage and compliance in Mexico with the two-dose and three-dose rotavirus vaccines
Source: Hum Vaccin Immunother. 2018 Dec 11;15(6):1251–9. doi: 10.1080/21645515.2018.1540827 (PMC6783135; doi:10.1080/21645515.2018.1540827)
Supplement: Supplemental Material [file khvi-15-06-1540827-s001.docx]

# Supplementary file 1

# Supplementary file 2

The following definitions were used for the calculation of outcomes.

| **Outcome** | ***Rotarix* 2010** | ***RotaTeq* 2012** | |
| --- | --- | --- | --- |
| **Vaccine coverage (%)** | | | |
| At least 1 dose coverage | (# infants with at least 1 dose) / (# infants <1y affiliated to IMSS) | | |
| Full series coverage | (# children receiving 2 doses) /  (# children <1y affiliated to IMSS) | | (# children receiving 3 doses) /  (# children <1y affiliated to IMSS) |
| **Vaccine compliance among vaccinated infants (%)** | | | |
| Full series completion | (# children receiving 2 doses) /  (# children with at least 1 dose) | | (# children receiving 3 doses) /  (# children with at least 1 dose) |
| Full series timeliness | (# children receiving 2 doses at correct age at first dose, interval between dose 1and 2, and age at last dose) /  (# children with at least 1 dose) | | (# children receiving 3 doses at correct age at first dose, interval between dose 1and 2, interval between dose 2 and 3, and age at last dose) /  (# children with at least 1 dose) |
| Timeliness: First dose given in week recommended | (# 1st dose given between 6-20 weeks of age) /  (# children with at least 1 dose) | | (# 1st dose given between 6-12 weeks of age) /  (# children with at least 1 dose) |
| Timeliness: Interval between first and second dose, and between second and third dose as recommended | (# 2nd dose given at least 4 weeks post dose 1) /  (# children with 2 doses) | | (# 2nd dose given 4-10 weeks post dose 1) /  (# children with at least 2 doses)  (# 3rd dose given 4-10 weeks post dose 2) /  (# children with 3 doses) |
| Timeliness: Last dose as recommended | (# 2nd dose given before 24 weeks of age) /  (# children with 2 doses) | | (# 3rd dose given before 32 weeks of age) /  (# children with 3 doses) |
| **Vaccine compliance among all eligible infants (%)** | | | |
| Full series completion timeliness | Same as full series timeliness, but with all vaccine-eligible infants as denominator | |  |

IMSS: Mexican Social Security Institute (Instituto Mexicano del Seguro Social); y: year; #: number

# Supplementary file 3

The four regions, based on the National Health and Nutrition Survey 2012 definitions, were:

▪ **North**: Baja California, Southern Baja California, Chihuahua, Coahuila, Durango, Nuevo Leon, Sonora, Sinaloa, Tamaulipas and Zacatecas;

▪ **Center**: Aguascalientes, Colima, Guanajuato, Hidalgo, Jalisco, State of Mexico, Michoacán, Nayarit, Querétaro, San Luis Potosí and Tlaxcala;

▪ **Mexico City**;

▪ **South**: Campeche, Chiapas, Guerrero, Morelos, Oaxaca, Puebla, Quintana Roo, Tabasco, Veracruz and Yucatán.

These regional definitions have been previously used in other epidemiological studies to compare results within the country.
